# Anti-Ige monoclonal antibody therapy for the treatment of chronic rhinosinusitis: a systematic review

**DOI:** 10.1186/1710-1492-10-S2-A19

**Published:** 2014-12-18

**Authors:** Chris J Hong, Adrian C Tsang, Jason Quinn, James Bonaparte, Adrienne Stevens, Shaun J Kilty

**Affiliations:** 1 Faculty of Medicine, University of Ottawa, Ottawa, ON, Canada; 2 Department of Pathology, Dalhousie University, Halifax, NS, Canada; 3 Department of Otolaryngology-Head and Neck Surgery, The University of Ottawa, The Ottawa Hospital, Ottawa, ON, Canada; 4 Center for Practice Changing Research, Ottawa Hospital Research Institute (OHRI), University of Ottawa, Ottawa, Canada

## Background

Several treatment options are available for chronic rhinosinusitis (CRS), but disease control remains elusive for many patients. Recently, literature has emerged describing anti-IgE monoclonal antibody as a potential therapy for CRS. However, its effectiveness and safety are not well known. The purpose of this systematic review was three-fold: to assess the effectiveness and safety of anti-IgE monoclonal antibody therapy for the treatment of adult patients with CRS and to identify evidence gaps to guide future research on anti-IgE monoclonal antibody therapy for the management of CRS.

## Methods

Methodology for the systematic review was registered with PROSPERO (No. CRD42014007600). A comprehensive literature search was performed of standard research databases, ClinicalTrials.gov and relevant grey literature sources. Only randomized controlled trials assessing anti-IgE therapy in adult patients for the treatment of CRS were included. Quality of evidence was evaluated using the GRADE approach. Two independent reviewers extracted data and discrepancies were settled by consensus and discussion amongst the reviewers.

## Results

Two studies met our inclusion criteria. The GRADE assessment of the quality of evidence was low. Comparison of anti-IgE therapy to placebo, there was significant differences in CT score and quality of life. There was a significant improvement in Lund-McKay score (n=1, 4.0 vs. -0.5, p=0.04) and AQLQ (n=1, 0.81 vs. 0.27, p=0.003). Mixed results were found for total nasal endoscopic polyp score in the two studies. No significant difference was seen with regards to nasal airflow and olfaction as measured by PNIF and UPSIT. No serious complications were reported with this therapy in either trial.

## Conclusions

Currently insufficient evidence exists to determine whether anti-IgE is more effective than placebo for the treatment of CRS. High quality studies are needed to supplement the evidence base in order to make a firm conclusion and to further assess anti-IgE monoclonal antibody therapy efficacy in this population.

